# Efficient Adhesion Culture of Human Pluripotent Stem Cells Using Laminin Fragments in an Uncoated Manner

**DOI:** 10.1038/srep41165

**Published:** 2017-01-30

**Authors:** Takamichi Miyazaki, Takehisa Isobe, Norio Nakatsuji, Hirofumi Suemori

**Affiliations:** 1 Institute for Integrated Cell-Material Sciences, Kyoto University, Kyoto 6068501, Japan; 2 Institute for Frontier Life and Medical Sciences, Kyoto University, Kyoto 6068507, Japan

## Abstract

We describe highly effective adhesion culture of human pluripotent stem cells (hPSCs) using laminin fragments without precoating. Culture substrates have been generally thought to exert a cell adhesion effect when they are precoated onto culture vessels. However, simple addition of laminin fragments to a cell suspension during passaging accelerated the adhesion of single dissociated hPSCs onto culture vessels that were not precoated with any culture substrate. Interestingly, similar to conventional precoating, the uncoated addition of laminin fragments supported robust adhesion of single hPSCs and maximum adhesion at a much lower concentration compared with precoating. Similar to precoating laminin fragments, hPSCs seeded with uncoated laminin fragments grew well without cell detachment and maintained pluripotency after continuous subculture. We tested other culture substrates, including full-length laminin and vitronectin, to support hPSC adhesion in the uncoated manner, but only laminin fragments had the potential for application in the uncoated manner. This cost-effective and time-efficient method may contribute to expansion of culture of hPSCs and accelerate the development of regenerative medicine using hPSCs.

Human pluripotent stem cells (hPSCs), including human embryonic stem cells (hESCs) and human induced pluripotent stem cells (hiPSCs), have an infinite proliferative potential and capacity for differentiation into all cell types of the three germ layers. It is highly expected that they will be used as cell sources for applications such as transplantation therapy and drug discovery, which are currently under development. To this end, stable expansion of undifferentiated stem cells is a fundamental technique. However, maintenance of hPSCs is still a complicated process that needs optimisation. Therefore, further improvements are required for efficient and stable expansion culture systems of hPSCs. Although there have been several approaches to optimise cultivation of hPSCs^1^, the modification of culture substrates used for coating the culture surface has greatly contributed to improving survivability after subculture and the expansion efficiency of hPSCs^2^,^3^. Such culture substrates for successful culture of undifferentiated hPSCs can be largely divided into two categories: synthetic substrates including Synthemax^4^ and PMEDSAH^5^, and recombinant protein-derivative culture substrates including laminins^2^,^3^,^6^ and truncated vitronectin^7^. Among these culture substrates, hPSCs show superior adhesion on specific recombinant protein derivatives compared with Matrigel™, which are widely used in the development of feeder-free and defined culture systems for hPSCs. We previously reported that specific laminin isoforms serve as applicable culture substrates for hPSC subculture^8^. Generally, cells have adhesion specificities for culture substrates, depending on the expression of cellular receptor integrins^9^. Undifferentiated hPSCs highly express laminin-binding-type integrin α6β1^2^,^8^ that has specific affinities for laminin-511, laminin-521, laminin-332, and laminin-111^10^. Indeed, hPSCs can be effectively cultured on these recombinantly produced laminin isoforms^3^,^6^,^8^. In addition, we have demonstrated that laminin fragments containing a minimal integrin-biding site enable superior adhesion and single cell-dissociated culture of hPSCs^2^. Currently, a recombinant laminin fragment based on laminin-511, commercialised as “iMatrix-511” (Nippi), has been widely used as a culture substrate in combination with various defined medium systems^11^,^12^,^13^,^14^,^15^. Use of recombinant proteins markedly improves stable maintenance of hPSCs. However, their use is a time-consuming and laborious process, because preparation of a culture surface using these substrates requires precoating for several hours before seeding cells.

In this study, we attempted to further improve the culture of hPSCs using the laminin fragment iMatrix-511, and found that addition of iMatrix-511 to the cell suspension was adequate during subculture, thereby omitting the procedure of precoating onto culture vessels. This finding was unexpected because hours of precoating culture substrates have been considered to be indispensable for adsorption onto culture vessels. In addition, uncoated use of iMatrix-511 showed comparable efficacy at a lower amount of the substrate than the precoating preparation of culture vessels. Finally, we demonstrate that iMatrix-511 can be efficiently applied in an uncoated manner to support long term maintenance of single cell-dissociated passaging of hPSCs. By applying uncoated iMatrix-511, the expansion of hPSCs may be more efficient and less costly, time consuming, and labour intensive.

## Results

### Adhesion efficiency of hPSCs cultured in the uncoated manner

The use of recombinant substrate proteins has improved the culture efficiency of undifferentiated hPSCs. However, it has been believed that precoating of the culture surface with these proteins is necessary before seeding cells. Because precoating is a time-consuming and laborious process, it would be beneficial to omit precoating. To address this issue, we examined whether substrate proteins work effectively when they are added to culture medium upon seeding cells. We used iMatrix-511, laminin-521, and rhVTN-N (truncated human vitronectin) as culture substrate proteins, because they are representative recombinant protein-derivative culture substrates that support undifferentiated culture of hPSCs and are commercially available. Although these culture substrates have been well characterised in the conventional precoated manner, they have not been evaluated in an uncoated manner, i.e. addition of culture substrates to the culture medium. Because extracellular matrix proteins vary considerably in protein size and charge, it is important to specifically optimise the concentration for each protein to support cell adhesion on culture vessels. We therefore initially estimated the dose dependency of culture substrates for hPSC adhesion and compared the maximum adhesion of hPSCs on the culture substrates by the different culture manners. The adhesion of hPSCs was evaluated in two defined media, TeSR-E8 as a low protein medium and StemFit AK03 as a protein-rich medium, to assess any inhibitory influence by the proteins in the culture medium on the adsorption of cultures substrates onto culture vessels.

**Figure 1:**
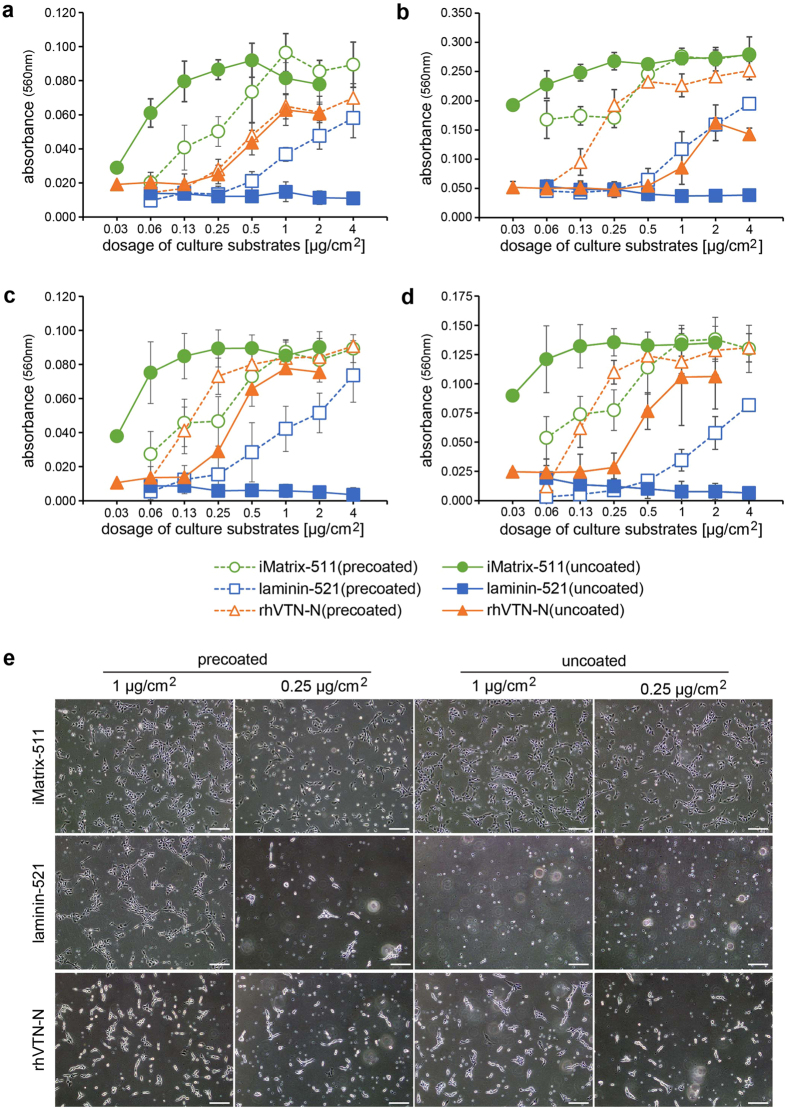
(**a**–**d**) Dose-response adhesion curves of hPSCs. hPSC adhesion in precoated and uncoated manners was estimated in two defined media as follows. (**a**) H9 hESCs in TeSR-E8 medium, (**b**) H9 hESCs in StemFit AK03 medium, (**c**) 253G1 hiPSCs in TeSR-E8 medium, and (**d**) 253G1 hiPSCs in StemFit AK03 medium. Data are the means of absorbance (560 nm) ±S.D. of five independent assays. hPSC adhesion at 1 μg/cm^2^ iMatrix-511 in uncoated usage was not significantly different from that in precoated usage [(**a**); *P* = 0.059, (**b**); *P* = 0.696, (**c**); *P* = 0.681, (**d**); *P* = 0.723], but significantly different at 0.25 μg/cm^2^ [(a); *P* < 0.001, (**b**); *P* < 0.001, (**c**); *P* < 0.001, (**d**); *P* < 0.001]. This result indicates that the use of iMatrix-511 is effective at a lower dosage in the uncoated manner. (**e**) Phase-contrast images of H9 hESCs cultured in precoated and uncoated manners. Images show the aspects of hPSC adhesion at 24 h after seeding in TeSR-E8 medium. Scale bar: 100 μm.

When single dissociated hPSCs were seeded in the conventional precoated culture manner, they adhered onto the three culture substrates in a dose-responsive manner as reported previously^2^,^3^,^7^ (Fig. 1a–d). In particular, hPSCs showed maximum adhesion on iMatrix-511 and comparable adhesion on rhVTN-N, although depending on the kind of defined medium. Laminin-521 supported slightly inferior adhesion compared with iMatrix-511 and rhVTN-N. On the other hand, the uncoated manner led to unexpected adhesion patterns of hPSCs. As expected, laminin-521 in the uncoated manner did not support hPSC adhesion even at a high concentration. rhVTN-N in the uncoated manner permitted hPSC adhesion at a high concentration, but the maximum adhesion of hPSCs was inferior to that in the precoated manner. However, unexpectedly, hPSCs showed maximum adhesion with iMatrix-511 in both uncoated and precoated manners. The maximum adhesion in the uncoated manner was equal to that in the precoated manner, but at a lower concentration (<0.25 μg/cm^2^) of the uncoated protein compared with the high concentration (>1 μg/cm^2^) of precoating, regardless of the culture medium and cell line.

Stable adhesion of hPSCs in the uncoated manner was also confirmed by the morphology of hPSCs at initial attachment. At the concentrations for maximum adhesion of hPSCs, i.e. 1 μg/cm^2^ in the precoated manner, hPSCs on each of the three culture substrates were flat and showed well elongated filopodia, indicating that they firmly attached to the substrates (Fig. 1e, Supplementary Fig. 1). hPSCs cultured in the uncoated manner with iMatrix-511 at 0.25 μg/cm^2^ showed similar morphology with flat and elongated filopodia compared with cells cultured in the precoated manner, suggesting that cell adhesion in the uncoated manner was similar to that in the precoating manner. In contrast, hPSCs cultured in the uncoated manner with laminin-521 did not show the adhesion even at a high concentration. Although rhVTN-N in the uncoated addition could support the adhesion of H9 hESCs in TeSR-E8 medium (Fig. 1e, Supplementary Fig. 1), hPSCs displayed poor adhesion at a high concentration in the uncoated addition of rhVTN-N, which were easily removed when exchanging the medium. These results indicated that the uncoated manner was only applicable to iMatrix-511, and hPSCs had superior adhesion at a lower concentration of iMatrix-511 in comparison with the conventional precoated manner.

**Figure 2:**
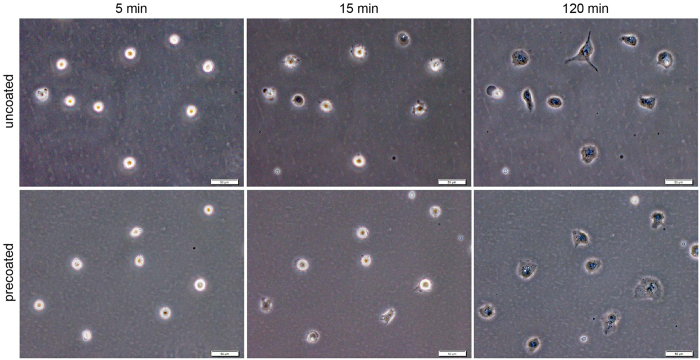
Serial Phase contrast images of H9 hESCs at initial adhesion in the uncoated manner. Note that hPSCs in the uncoated manner showed robust adhesion as early as that in the precoated manner.

We next confirmed the stability of hPSC adhesion in the uncoated manner by observing initial adhesion in dynamic images. hPSCs cultured in the precoated manner rapidly adhered and elongated soon after seeding (Fig. 2). In addition, similar to the immediate adhesion rate in the precoated manner, hPSCs cultured in the uncoated manner showed strong adhesion with morphological changes after seeding. Conversely, hPSCs cultured in the uncoated manner without iMatrix-511 had a curled shape without specific adhesion. These observations indicated that hPSCs adhered onto culture vessels in the uncoated manner at the same rate and adhesion strength as those in the standard precoated manner, and only addition of iMatrix-511 to the culture medium supported rapid and stable adhesion.

**Figure 3:**
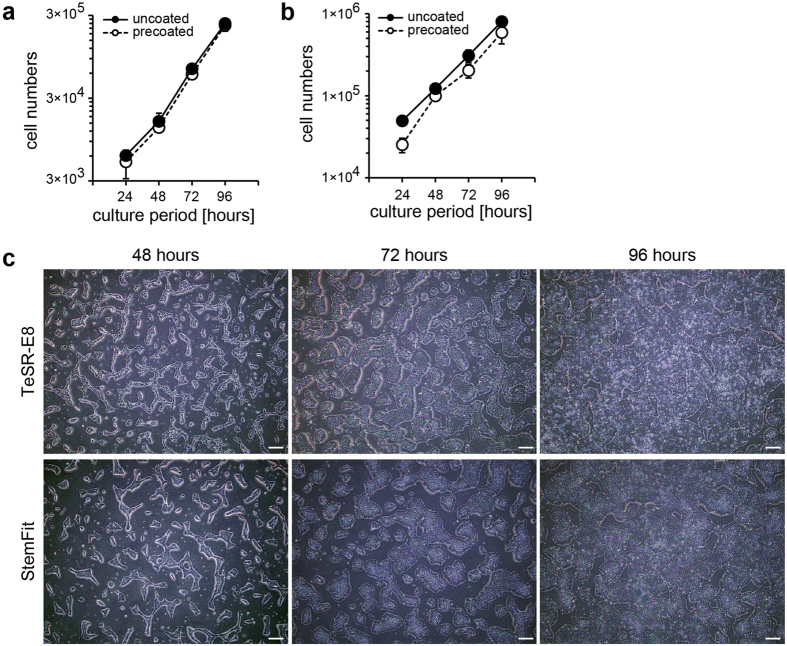
(**a**,**b**) Short-term growth curves of hPSCs in each culture manner. (**a**) H9 hESCs in TeSR-E8 medium and (**b**) H9 hESCs in StemFit AK03 medium. hPSCs cultured in the uncoated manner proliferated and had a similar growth rates as those in the precoated manner. (**c**) Phase-contrast images of H9 hESCs in TeSR-E8 and StemFit AK03 medium. Similar to the precoated manner, cells cultured in the uncoated manner proliferated normally without any colony detachment. Scale bar: 100 µm.

We then evaluated whether the uncoated manner supports steady adhesion of hPSCs for long periods. The evaluation of hPSC growth was performed under the conditions for maximum cell adhesion (0.25 μg/cm^2^ iMatrix-511 for the uncoated manner and 1 μg/cm^2^ for the precoated manner). hPSCs seeded in both manners showed superior adhesion at nearly the same maximum levels, and they proliferated at similar rates (Fig. 3a and b, Supplementary Fig. 2a and b). hPSCs cultured in the uncoated manner showed a standard hPSC morphology, suggesting maintenance of the undifferentiated cellular state (Fig. 3c, Supplementary Fig. 2c). These results indicated that the uncoated manner supports superior cell adhesion similar to the precoated manner without detachment during proliferation.

### Characterisation of hPSCs grown in the uncoated manner

**Figure 4:**
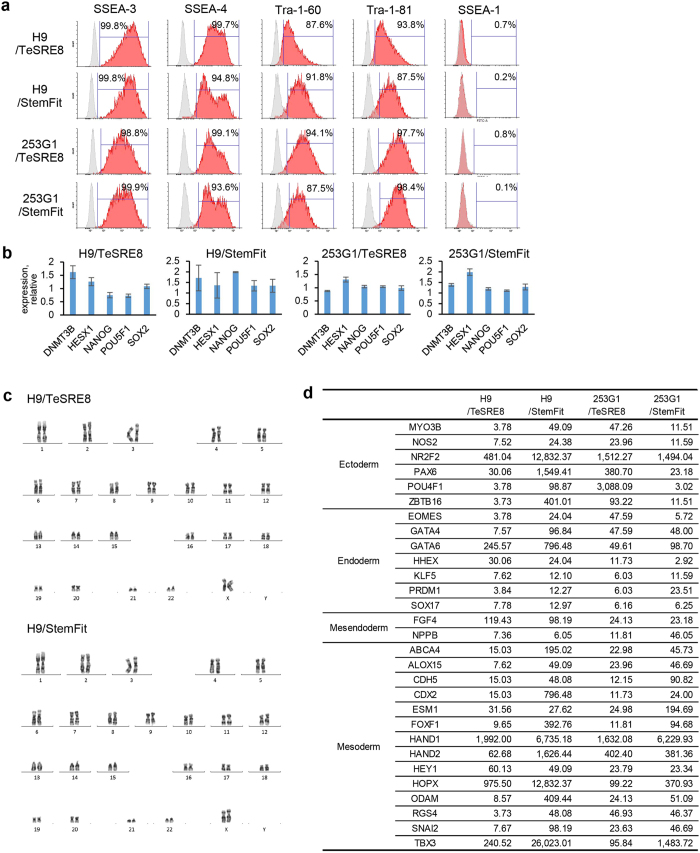
Characterisation of hPSCs grown in the uncoated manner. (**a**) Flow cytometric analysis of representative undifferentiated markers. Gray shaded area shows the negative control. (**b**) Quantitative PCR analysis of undifferentiated marker genes. Graph shows relative expression of hPSCs grown for 29 passages in the uncoated manner compared with those grown for 10 passages in the precoated manner. (**c**) Normal karyotypes analysed by G-banding. H9 hESCs had a normal karyotype (46, XX). (**d**) Quantitative PCR analysis of marker genes for the differentiation of embryoid bodies. hPSCs were cultured in the uncoated manner and then induced to form embryoid bodies for 14 days. Expression of representative differentiation marker genes was compared with that before embryoid body formation by analysing a single data set of the TaqMan hPSC Scorecard Panel system, and gene sets showing a more than two-fold increase in expression are listed. Data were collected from hPSCs grown for 10 passages in the uncoated manner.

We next estimated the normality of hPSCs after long term culture in the uncoated manner. hPSCs were continuously passaged in the uncoated manner with iMatrix-511 for more than 10 passages. Similar to cells cultured in the precoated manner, hPSCs grown in the uncoated manner proliferated well and maintained a typical cell shape. In addition, they highly expressed representative undifferentiated cellular surface markers SSEA-3, SSEA-4, Tra-1-60, and Tra-1-81, and did not express SSEA-1, indicating a sustained undifferentiated state similar to that of cells grown in the conventional precoated manner (Fig. 4a). Quantitative PCR analysis of hPSCs grown in the uncoated manner also showed the undifferentiated state of hPSCs. They sustained similar expression levels of undifferentiated marker genes compared with the precoated manner (Fig. 4b), indicating little difference in the undifferentiated state of hPSCs grown in uncoated or precoated manners. Karyotypic analysis revealed that the uncoated manner did not affect the stability of hPSCs (Fig. 4c, Supplementary Fig. 3). These results indicated that the uncoated manner sustained the undifferentiated state of hPSCs even after continuous subculture. Lastly, we estimated the differentiation potential of hPSCs grown in the uncoated manner for multiple cell lineages. The formation of embryoid bodies by hPSCs was normal. By comparing gene expression before induction, the embryoid bodies had highly increased expression of marker genes specific for the three germ layers, indicating that they maintained their differentiation potential (Fig. 4d). Thus, we conclude that hPSCs grown in the uncoated manner sustain their pluripotency, and the uncoated manner can be applied to continuous daily subculture of hPSCs.

## Discussion

We demonstrated undifferentiated culture of hPSCs with iMatrix-511 in an uncoated manner that omits the precoating process of the culture substrate required in advance of passaging. In addition, this uncoated manner markedly reduced the amount of substrate needed for the maximum adhesion of hPSCs compared with the precoated manner.

Similar to other adhesion-dependent cultured cells, hPSCs require suitable substrates to maintain their undifferentiated state^16^. It has been believed that culture substrates need to be precoated for 1 to several hours prior to passaging to ensure adequate absorption of substrates to the culture surface. Therefore, it would be less laborious if a prepared culture surface is readily available. However, the culture substrates widely used for feeder-free culture of hPSCs, such as vitronectin, laminin-521, and iMatrix-511, cannot be stored at ambient conditions after coating.

Therefore, we investigated other approaches to use culture substrates with less labour. We unexpectedly found that simple addition of iMatrix-511 to the culture medium upon seeding cells during passaging was sufficient to support cell adhesion to the culture surface. Furthermore, a much lower amount of iMatrix-511 (0.13–0.25 μg/cm^2^; ca 5–10 times less than the precoated manner) was effective for maximum adhesion compared with the precoating method.

Because culture medium generally contains large amounts of proteins, such as serum albumins, which are thought to disturb absorption of substrates by blocking or masking culture vessels^17^,^18^, uncoated usage of culture substrate proteins is usually inefficient for cell adhesion. Indeed, full-length laminin-521 supported superior adhesion of hPSCs in the precoated condition, but it did not support hPSC adhesion in the uncoated manner as expected. However, similar results were obtained when using a low protein medium, TeSR-E8, with laminin-521. Conversely, rhVTN-N and iMatrix-511 supported comparable adhesion of hPSCs in both precoated and uncoated manners regardless of the medium type, even though rhVTN-N in the uncoated manner required a much higher amount than in the precoated manner. Hence, the presence of a high protein concentration in the medium does not affect the efficacy of the substrate in the uncoated manner, but the properties of the substrate protein may be crucial for use in the uncoated manner.

The small molecular size of iMatrix-511 could account for this phenomenon, because rhVTN-N in the uncoated manner was effective even though it required a much higher concentration than in the precoated manner in most cases. Small proteins in suspension can easily access receptors for substrate proteins on the whole cell surface, and might support adhesion to the culture surface more effectively. Although it is unclear why only iMatrix-511 is effective at a much lower concentration than in the precoated manner, this particular feature of iMatrix-511 makes it beneficial for expansion of hPSCs.

In conclusion, iMatrix-511 effectively support cell adhesion when added as a medium supplement and not applied by precoating. Unlike other substrate proteins examined, uncoated iMatrix-511 supports cell adhesion and long-term expansion at a much lower concentration compared with the precoated manner. This new method of using iMatrix-511 would allow less costly and time-consuming maintenance of hPSCs.

## Methods

### Precoating of culture substrates

For the dose–response adhesion assay, 96-well flat-bottomed plates (cell culture treated; BD Falcon) were precoated with iMatrix-511 (Nippi), laminin-521 (Biolamina), or rhVTN-N (20–398 aa of vitronectin; Wako) at concentrations of 0–4 μg/cm^2^ for 3 h at 37 °C in a CO_2_ incubator just prior to cell seeding. For adhesion efficiency assays and passaging for comparison with the uncoated manner, 24- or 6-well plates (BD Falcon) were coated with iMatrix-511 at 1 μg/cm^2^. Dulbecco’s phosphate buffered saline (DPBS) was used to dissolve the culture substrates. The solution volume for coating was standardised to 100 μl/cm^2^, but modified to 50 μl/well for 96-well plates.

### Standard cell culture

The hESC line H9 and hiPSC line 253G1 were maintained in defined culture media TeSR-E8 (StemCell Technologies) and StemFit AK03 (Ajinomoto). Every 4 days at confluency, the cells were passaged by treatment with a detachment solution consisting of 0.5× TrypLE select (Life Technologies) and 5 mM EDTA/DPBS for 5 min at room temperature. The cells were seeded at 2 × 10^4^ cells/cm^2^ onto culture vessels either in a precoated or uncoated manner as described below. After passaging, the culture medium without Y-27632 or any culture substrate was changed daily both in the precoated and uncoated manners. The volume of culture medium applied to culture vessels was standardised to 200 μl/cm^2^. The use of hESC lines were performed in conformity with “The Guidelines on the Distribution and Utilization of Human Embryonic Stem Cells” of the Ministry of Education, Culture, Sports, Science and Technology, Japan.

### Cell culture in the precoated manner

hPSCs were seeded in culture medium containing 10 μM Y-27632 onto culture vessels coated with culture substrates as described above. After seeding, hPSCs were subcultured as described above.

### Cell culture in the uncoated manner

hPSCs were seeded in culture medium containing 10 μM Y-27632 and culture substrates. For general culture in the uncoated manner using iMatrix-511, the concentration of iMatrix-511 was 0.25 μg/cm^2^. For example, 5 μl of 0.5 mg/ml iMatrix-511 stock solution was added into 2 ml of cell suspension for culture in one well of a 6-well plate without any coating. After seeding, hPSCs were subcultured as described in *Standard cell culture*.

### Cell adhesion assay

hPSCs, which were maintained in the precoated manner, were detached as described above and seeded at 5 × 10^4^ cells/cm^2^ in defined medium containing 10 μM Y-27632 with or without the culture substrates at 0–4 μg/cm^2^ according to the culture manner (precoated or uncoated). After 24 h of incubation, non-adherent cells were carefully removed by rinsing the plates once with warm Dulbecco’s modified Eagle’s medium (DMEM)/F12. The remaining adherent cells were fixed with 10% formalin for 15 min and then postfixed in 100% ethanol for 5 min at room temperature. The cells were stained with 10% Giemsa in MilliQ water for 1 h at room temperature. After extensive washing with MilliQ water and drying the plates, the remaining cells were solubilised by addition of 1% SDS. The absorbance was then measured at 560 nm by a multiwell plate reader.

### Cell proliferation assay

hPSCs, which were maintained in the precoated manner, were seeded at 2 × 10^4^ cells/cm^2^ into a 24-plate for H9 hESCs or 6-well plate for 253G1 hiPSCs by the precoated or uncoated manner. Viable H9 hESCs estimated by trypan blue staining were counted every 24 h. Viable 253G1 hiPSCs were counted by a NucleoCounter N-200 every 24 h.

### Flow cytometric analysis

Cells were dissociated by treatment with 5 mM EDTA/DPBS for 3 min, followed by further treatment with a 0.05% trypsin/EDTA solution for 1 min. After two washes with DMEM/10% foetal bovine serum and one wash with staining buffer (0.1% bovine serum albumin/DPBS), 1 × 10^5^ cells were incubated for 30 min at 4 °C with primary antibodies diluted in staining buffer. The cells were then rinsed twice with staining buffer and incubated for 30 min at 4 °C with secondary antibodies diluted in staining buffer. Then, the cells were rinsed twice with staining buffer and counterstained with propidium iodide just prior to analysis. Fluorescence intensities were analysed on a FACSCanto flow cytometer (Becton Dickinson), and the FL-1 positive ratios in the propidium iodide-negative region were monitored using FACSDiva software. Primary antibodies against the following molecules were used: stage-specific embryonic antigen (SSEA)-3 [2 μg/ml; Developmental Studies Hybridoma Bank (DSHB)], SSEA-4 (1 μg/ml; DSHB), Tra-1-60 (1 μg/ml; Millipore), Tra-1-81 (1 μg/ml; Millipore), and SSEA-1 (2 μg/ml; DSHB). Anti-mouse Ig/FITC conjugated (10 μg/ml; Becton Dickinson) and anti-rat IgM/Alexa 488 conjugated (1 μg/ml; Molecular Probes) were used as secondary antibodies.

### Quantitative real-time PCR

The undifferentiated state after subculture in each culture manner was estimated by quantitative real-time (qRT)-PCR. Total RNA was isolated using an RNeasy Micro Kit (QIAGEN), and cDNA was synthesized using a ReverTra Ace qPCR RT Kit (TOYOBO) according to the manufacturers’ instructions. qRT-PCR assays were performed using the Applied Biosystems StepOnePlus realtime PCR system (Applied Biosystems) with Fast SYBR Green Master Mix (ThermoFisher Scientific). All reactions were performed in triplicate with 10 ng cDNA. The relative expression of undifferentiated marker genes was estimated with GAPDH expression as the reference. The primers used in qRT-PCR are listed in Supplementary Table 1. To demonstrate the stable culture manner of uncoated usage, the expression ratio of hPSCs subcultured in the uncoated manner for 29 passages was calculated by comparing with the gene expression in the precoated manner for 10 passages.

### Differentiation assay

The differentiation potential of subcultured hPSCs was estimated by forming embryoid bodies. To form embryoid bodies, hPSCs were first treated with CTK solution consisting of 1 mg/ml collagenase IV (Life Technologies), 0.25% trypsin (Life Technologies), 1 mM CaCl_2_, and 20% knockout serum replacement. The cells were detached as clumps using a cell scraper and then cultured as a suspension in petri dishes containing hESC medium without fibroblast growth factor-2. The medium was changed every 2 days. After 14 days, total RNA was extracted using an RNeasy Micro Kit (Qiagen), and the expression levels of differentiation markers were estimated by quantitative PCR. The quantitative PCR was performed according to the instructions of the TaqMan hPSC Scorecard Panel (ThermoFisher Scientific). Expression of differentiation marker genes in embryoid bodies was evaluated by comparing the single data set of the TaqMan hPSC Scorecard Panel against that in undifferentiated hPSCs.

### Karyotyping

hPSCs grown for 10 passages in the uncoated manner were treated with 100 ng/ml colcemid (Life Technologies) for 2 h. After dissociation in 0.25% trypsin/EDTA, the cells were treated with a hypotonic solution and then fixed in Carnoy’s solution. The cells were spread onto glass slides and stained with Giemsa. Chromosome spreads were analysed by randomly counting 30 cells using the Ikaros Karyotyping System (META system).

### Statistical analysis

Statistical differences were determined by the two-sided paired *t*-test. Differences of *P* < 0.05 were considered significant.

## Additional Information

**How to cite this article**: Miyazaki, T. *et al*. Efficient Adhesion Culture of Human Pluripotent Stem Cells Using Laminin Fragments in an Uncoated Manner. *Sci. Rep.* **7**, 41165; doi: 10.1038/srep41165 (2017).

**Publisher's note:** Springer Nature remains neutral with regard to jurisdictional claims in published maps and institutional affiliations.

## Supplementary Material

Supplementary Information
