# Supplementary material for: Efficient Adhesion Culture of Human Pluripotent Stem Cells Using Laminin Fragments in an Uncoated Manner
Source: Sci Rep. 2017 Jan 30;7:41165. doi: 10.1038/srep41165 (PMC5278368; doi:10.1038/srep41165)
Supplement: Supplementary Information [file srep41165-s1.pdf]

Supplementary Information

Efficient Adhesion Culture of Human Pluripotent Stem Cells Using Laminin Fragments in an Uncoated Manner

Takamichi Miyazaki[1], Takehisa Isobe[1], Norio Nakatsuji[1,2], and Hirofumi Suemori[2,*]

[1] Institute for Integrated Cell-Material Sciences, Kyoto University, Kyoto 6068501, Japan
[2] Institute for Frontier Life and Medical Sciences, Kyoto University, Kyoto 6068507, Japan

* Correspondence should be addressed to H.S. (hsuemori@frontier.kyoto-u.ac.jp)

Supplementary Figures 1 - 3

Supplementary Table 1

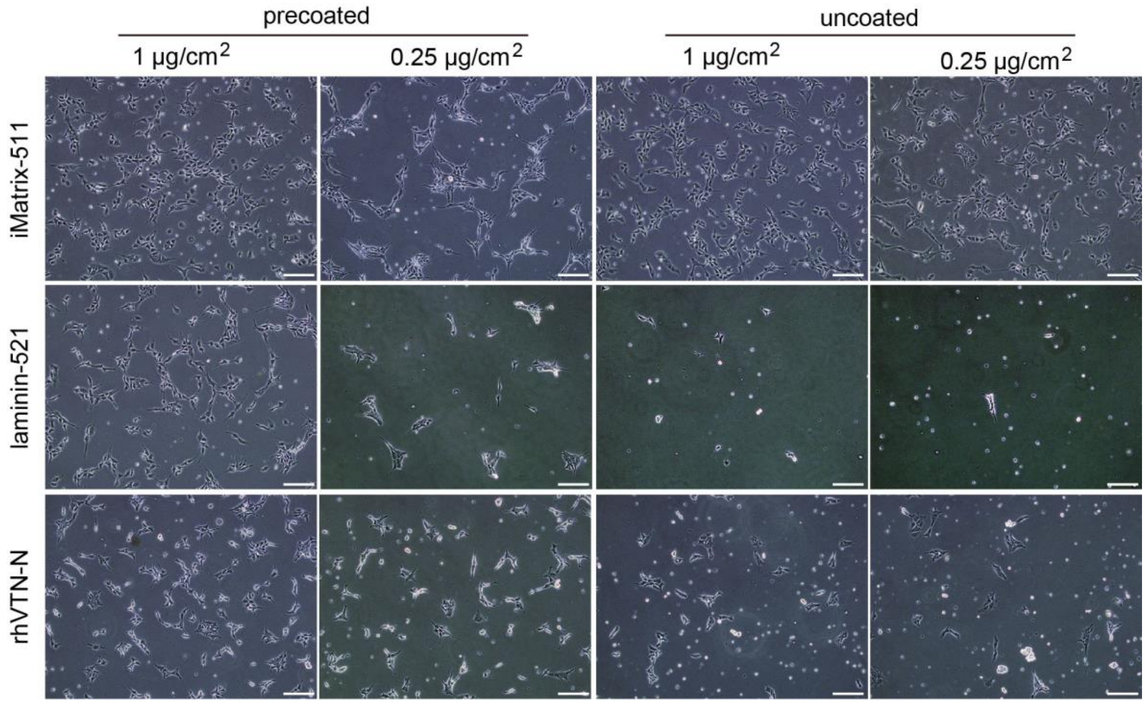

Supplementary Figure 1

Phase-contrast images of 253G1 iPSCs cultured in precoated and uncoated manners. Images show the aspects of hPSC adhesion at 24 h after seeding in TeSR-E8 medium. Scale bar: 100 µm.

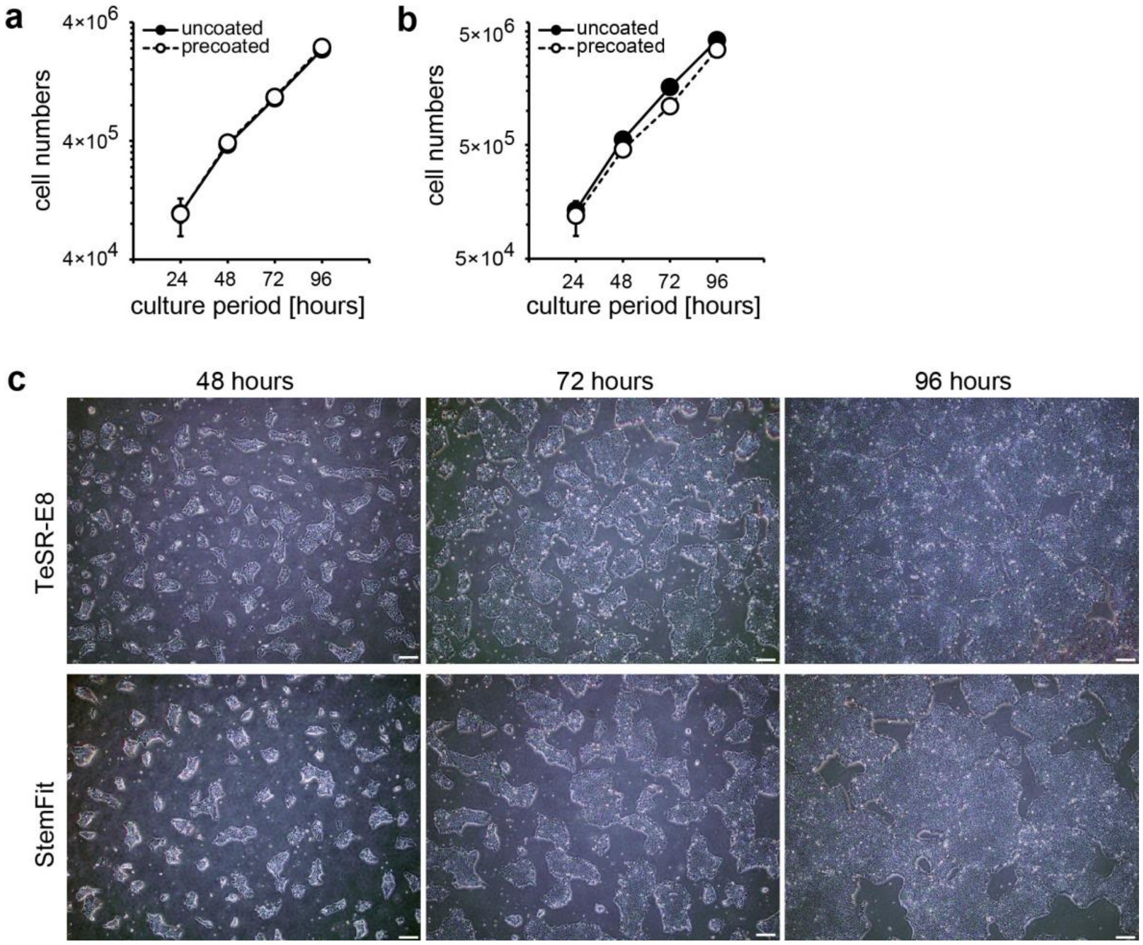

Supplementary Figure 2

(**a**, **b**) Short term growth curves of hPSCs in each culture manner. (**a**) 253G1 iPSCs in TeSR-E8 medium and (**b**) 253G1 hiPSCs in StemFit AK03 medium.

(**c**) Phase-contrast images of 253G1 hiPSCs in TeSR-E8 and StemFit AK03 medium cultured in the uncoated manner. Scale bar: 200 μm.

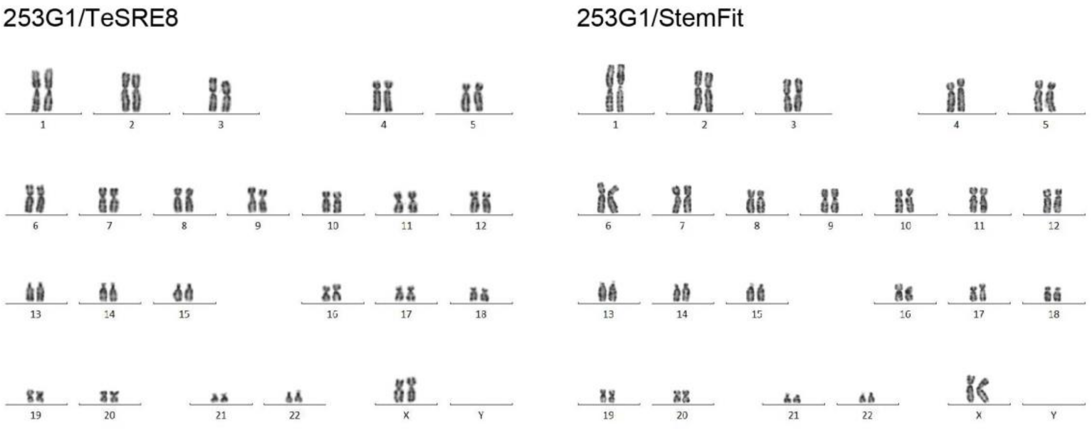

Supplementary Figure 3

Karyotypes analysed by G-banding. 253G1 iPSCs had a normal karyotype (46, XX).

Supplementary Table 1: qPCR primer sequences.

Primer	Forward Primer 5’->3’	Reverse Primer 5’->3’
DNMT3B	ACCTCGTGTGGGGAAAGATCA	CCATCGCCAAACCACTGGA
HESX1	GCGTGGTGGATCACCCAAT	CGGCCTCTATACCAACTCAACT
NANOG	CCCCAGCCTTTACTCTTCCTA	CCAGGTTGAATTGTTCCAGGTC
POU5F1	ACATCAAAGCTCTGCAGAAAGAACT	CTGAATACCTTCCCAAATAGAACCC
SOX2	GCCGAGTGGAAACTTTTGTCG	GGCAGCGTGTACTTATCCTTCT
GAPDH	GAAGGTGAAGGTCGGAGTC	GAAGATGGTGATGGGATTTC
